# Performance of the Techcyte AI-based image analysis system for coproparasitological diagnosis in clinical stool specimens: a retrospective evaluation study

**DOI:** 10.1128/jcm.00431-26

**Published:** 2026-06-22

**Authors:** Carles Rubio Maturana, Elena Sulleiro, Francesc Zarzuela, Patricia Martínez-Vallejo, Alejandro Mediavilla, Aroa Silgado, Carlos Turró, Martha Balladares, Ana Gracia, Carmen Paz, Daniel García-Vega, Sol María San José-Villar, Albert Blanco-Grau, Fernando Moreno, Nieves Larrosa, Lidia Goterris

**Affiliations:** 1 Microbiology Department, Vall d’Hebron University Hospital, Vall d’Hebron Research Institute (VHIR) 203275, Barcelona, Spain; 2 Department of Microbiology and Genetics, Universitat Autònoma de Barcelona (UAB) 16719, Barcelona, Spain; 3 CIBERINFEC, ISCIII-CIBER de Enfermedades Infecciosas, Instituto de Salud Carlos III, Madrid, Spain; 4 Clinical Biochemistry Department, Vall d'Hebron University Hospital 16810 , Barcelona, Spain; 5 Clinical Biochemistry, Drug Delivery and Therapy Research Group, Vall d'Hebron Research Institute, Barcelona, Spain; Mayo Clinic Minnesota, Rochester, Minnesota, USA

**Keywords:** coproparasitological diagnosis, microscopic examination, artificial intelligence, protozoa, helminths

## Abstract

Coproparasitological stool analysis based on microscopic examination is the reference diagnostic technique routinely performed in clinical microbiology laboratories. Automated image analysis systems could offer a suitable solution, reducing technologist workload and improving screening efficiency. The Human Fecal Ova & Parasite (O&P) Detection Solution (Techcyte) is an artificial intelligence (AI) software employing imaging-based algorithms to provide screening presumptive diagnostic results. This study aimed to validate the Wet Mount Iodine Solution software for detecting and diagnosing protozoan and helminthic infections in stool specimens. A total of 178 clinical stool specimens were retrospectively analyzed between July and October 2024 for comparative evaluation of the AI-based system. Positive specimens with confirmed mono (*n* = 116) and mixed (*n* = 31) protozoa and helminth infections, as well as negative specimens (*n* = 31), were included. Conventional microscopy results were compared with AI presumptive detection and identification outcomes. In addition, operator-reviewed AI results were evaluated against the reference diagnosis. The system achieved 96.62% positive percent agreement (PPA) and 93.33% negative percent agreement (NPA) for overall detection considering operator-reviewed interpretation. The kappa coefficient was 0.865, indicating strong agreement with conventional microscopy. The AI system detected additional parasites in 74/178 (41.57%) specimens, providing a diagnostic improvement compared to traditional microscopy. AI-assisted microscopy enhances intestinal parasite diagnosis, providing reliable results when combined with operator review. Despite limitations such as specimen low volume analysis and artifact detection, the system shows strong potential for improving diagnostic workflows in clinical microbiology laboratories.

**IMPORTANCE:**

Microscopic examination of stool specimens remains the reference technique for diagnosing intestinal parasitic infections although it is labor-intensive and operator-dependent methodology. The implementation of artificial intelligence-assisted microscopy offers a promising approach to streamline diagnostic workflows, improve consistency, and enhance traditional methods. Our evaluation study provides valuable clinical evaluation data of the Human Fecal Ova & Parasite (O&P) Detection Wet Mount Iodine Solution (Techcyte), demonstrating high sensitivity, specificity, and agreement with conventional microscopy. Importantly, AI-assisted review yielded diagnostic gains in a substantial proportion of specimens, underscoring its value as a screening and complementary tool for routine parasitological diagnostics. These findings highlight the potential of AI-powered image analysis to improve the accuracy and efficiency of coproparasitological testing, thereby supporting clinical decision-making and optimizing resource utilization in microbiology laboratories.

## INTRODUCTION

Intestinal parasitic infections pose a significant global health threat. Worldwide, to diagnose intestinal parasitic infections, stool examination remains a crucial tool in clinical laboratories. Traditional manual methods, such as direct microscopic examination after concentration techniques of wet smear, are the most commonly employed for parasite detection (1) although these methods vary in sensitivity and processing time (2). While direct microscopy examination methods are widely used due to their simplicity and cost-effectiveness, they are expert-dependent, time-consuming, labor-intensive techniques that require high levels of expertise and represent a high workload for clinical laboratory routine.

In response to these challenges, automated digital imaging systems based on artificial intelligence (AI) for stool examination have emerged as promising tools to improve the efficiency and accuracy of object detection (3–5). Automated AI-based examination is already being employed for hematological, urinary, culture, and cytology specimens to support clinical laboratories and implemented as a reinforcing tool for classical microscopy techniques (6). However, stool specimen examination is still a challenge for imaging system devices, and several developmental and validation studies are demonstrating the AI potential for this aim (3–5). The high number of artifacts and confusing forms in stool specimens are a handicap for object detection systems; therefore, operator review interpretation is crucial to obtain reliable results. Early validation of such systems highlights the potential of AI-assisted microscopy to reduce the need for labor-intensive microscopy, paving the way toward high-throughput and cost-effective diagnostic methods. This study presents the initial clinical evaluation of the Human Fecal Ova & Parasite (O&P) Detection Wet Mount Iodine Solution (Techcyte, Inc., Orem, Utah, United States of America) designed to detect and identify protozoa and helminths in stool specimens using wet mount preparation with Lugol-based stain (Wet Mount Iodine Solution). The system acts as a high sensitive scanning tool to perform a pre-diagnosis before operator review of detected objects through an AI-based platform.

## MATERIALS AND METHODS

### Study design

A retrospective comparative evaluation study was performed between July and October 2024 based on Clinical Microbiology validation guidelines (7). The study was conducted using a convenience specimen collection consisting of stored fixed and concentrated clinical stool specimens from patients previously diagnosed with intestinal parasites at Drassanes—Vall d’Hebron International Health and Communicable Diseases Centre (DVH), Vall d’Hebron University Hospital (VHUH), and primary care centers in Barcelona, Spain. Results from conventional microscopy examination were compared with the AI system (Wet Mount Iodine Solution) detections and identifications. The Techcyte Wet Mount Iodine Solution is an AI-assisted screening tool designed to help medical technologists locate, count, and pre-classify parasites from iodine-stained fecal slides (8). Positive specimens with confirmed mono (*n* = 116) and mixed (*n* = 31) infections, containing protozoa and helminths as detailed in Table 1, were included in the study, along with negative specimens (*n* = 31). Two specimens containing *Dicrocoelium dendriticum* and *Schistosoma intercalatum* were analyzed to evaluate potential detection, but not specific identification, as the software does not have these aetiologies categorized; consequently, these specimens were excluded from the general comparative evaluation analysis.

**TABLE 1:** Parasitic etiology included in the study

Protozoa	Helminths
*Blastocystis* species	*Schistosoma mansoni*
*Iodamoeba bütschlii*	*Enterobius vermicularis*
*Giardia duodenalis*	*Dicrocoelium dendriticum^a^*
*Entamoeba coli*	*Trichuris trichiura*
*Entamoeba hartmanni*	*Hymenolepis/Rodentolepis nana*
*Entamoeba* species	*Hookworm*
*Entamoeba histolytica/dispar*	*Strongyloides stercoralis*
*Endolimax nana*	*Ascaris lumbricoides*
*Dientamoeba fragilis*	*Cystoisospora belli*
*Chilomastix mesnili*	*Taenia species*
*Balantioides coli*	*Diphyllobothrium latum*
	*Fasciola hepatica*
	*Hymenolepis diminuta*
	*Schistosoma intercalatum^a^*

^*a*^ These etiologies do not represent categories that can be specifically identified by the AI-software.

### Specimen preparation for conventional microscopy examination

Specimens from primary care centers and VHUH were self-collected by patients and immediately fixed into the Midi Parasep (9). One gram of feces collected with the spoon of the kit was added to 8 mL of Alcorfix fixative contained on the mixing chamber. Once at the laboratory, the Midi Parasep vials were vortexed and centrifuged at 2,302 × *g* for 3 min. Specimens from DVH were prepared following the Ritchie technique (formol-ether) (10). After decanting the supernatant, one drop of the pellet was stained with one drop of saline-Lugol on a slide and covered with a 24 × 60 mm coverslip. Slides were examined under the microscope at 100× and 400× magnification. When there was suspicion of helminth infection (based on patient’s epidemiological context and/or presence of elevated eosinophilia [>500 eosinophils/µL]), the test was repeated three times or until the pellet was depleted. Protozoa and helminths were identified according to morphological characteristics via microscopic examination as considered the reference diagnostic method (11).

### Specimen preparation for scanning

**Fig 1:**
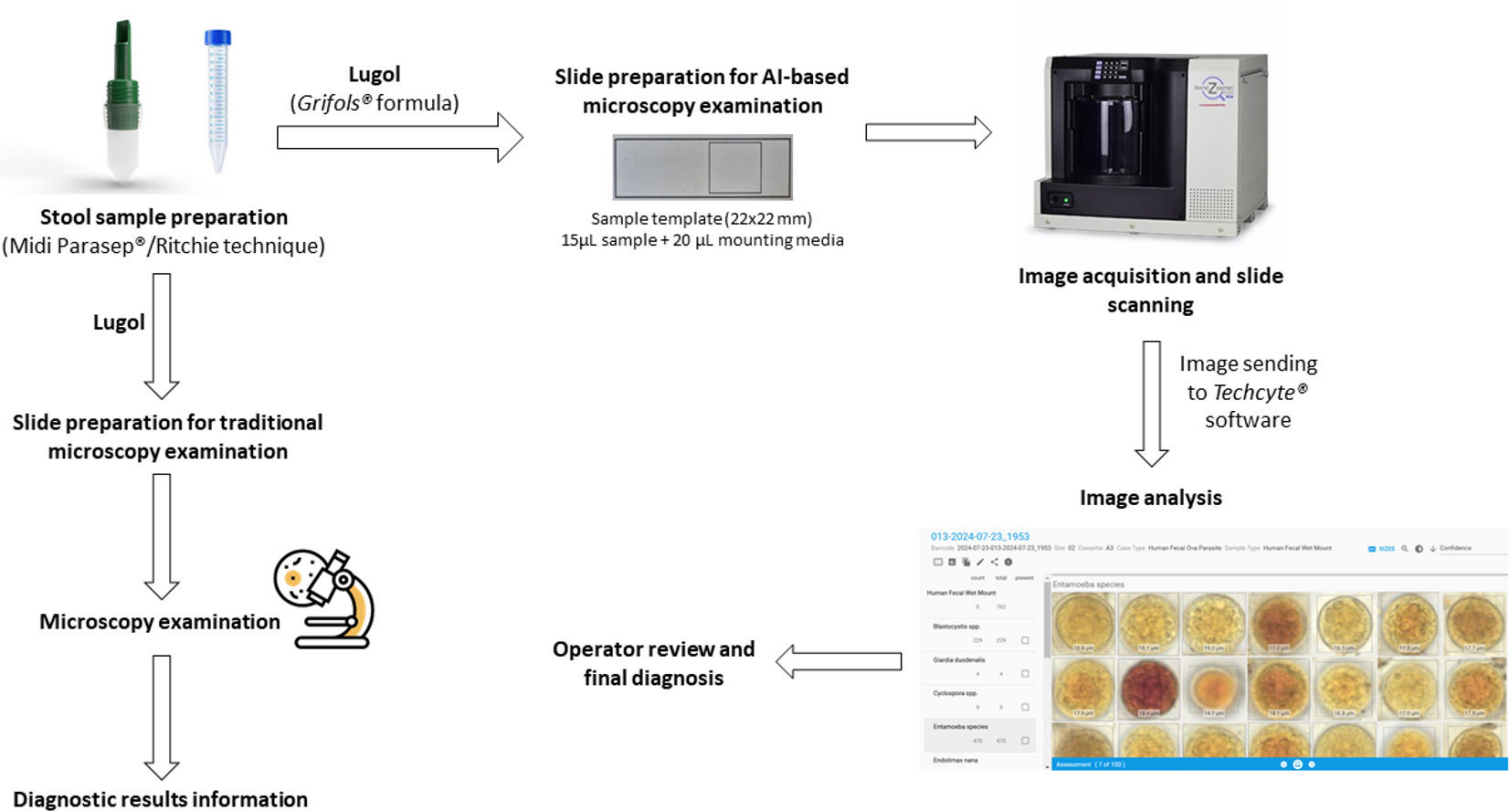
Operational flowchart of traditional microscopy examination of stool specimens and AI-based examination Techcyte solution procedure.

The same stool pellets obtained for routine microscopic examination were employed for scanning and acquiring the images for AI software analysis. All retrospective specimens were examined by conventional microscopy before its preparation for AI analysis. The same specimen was employed by both microscopy techniques although a different slide was processed for each. A total of 15 µL of stool pellet were mixed with 20 µL of Lugol-based mounting media (850 mL of Clinical Laboratory Reagent Water, 80 g of Iodine 99.8%, and 160 g of Potassium Iodide) and placed within a 22 × 22 mm squared template area on a glass microscope slide (pre-cleaned, 1.0 mm–1.2 mm thick, 26 × 76 mm double frosted, 45° corners, ground edges) (Fig. 1). A coverslip (20 × 20 mm) was placed also on the squared area after specimen displacement, and the slides were introduced into the NanoZoomer S360MD Slide Scanner System (Hamamatsu Photonics, Japan) for scanning.

### Slide scanning and image analysis

A high-resolution image (jpeg, 18.5 × 18.5 mm, 80,640 × 80,640 pixels, 110,434 DPI) was acquired by the NanoZoomer S360MD Slide Scanner for each specimen, with two Z-stacks per specimen to avoid focus issues by internal Hamamatsu Quality Checking (QC) Mode (12). Image quality and fidelity are ensured through the system’s integrated NZAcquireMD software, which utilizes a pre-focus map with five focus points per 15 mm × 15 mm area to maintain optimal color balance and a high-resolution scanning performance of approximately 0.23 µm/pixel. Furthermore, the system incorporates a dedicated QC mode that empowers the operator to verify focus and image quality parameters prior to final image generation (12, 13). The scanner allows batch loading of slides although not continuous loading. For each slide, the image capture time was 2 min and 45 s. Once acquired, images were sent automatically via internet connection to Techcyte server for AI image analysis, and results were showed in Techcyte software platform for operator review. The AI platform is designed to detect a broad range of intestinal pathogens, categorized by the system into specific classes or aggregate groups based on morphological criteria. The detectable helminths include: *Ascaris lumbricoides*, *Clonorchis sinensis/Opisthorchis* spp., *Enterobius vermicularis*, *Fasciola* spp.*/Fasciolopsis buski*, *Diphyllobothrium latum* (Fish Tapeworm), *Hookworm/Trichostrongylus*, *Hymenolepis diminuta*, *Paragonimus* spp., *Paracapillaria philippinensis*, *Rodentolepsis nana* (*Hymenolepis nana*), *Schistosoma mansoni*, *Schistosoma japonicum/S. mekongi*, *Strongyloides* spp., *Taenia* spp., and *Trichuris trichiura*. Regarding protozoa, the system identifies *Balantioides coli*, *Blastocystis* spp., *Chilomastix mesnili*, *Cyclospora* spp., *Cystoisospora belli*, *Endolimax nana*, *Entamoeba* species (includes *Entamoeba coli*, *Entamoeba histolytica/dispar*, and *Entamoeba polecki*), *Giardia duodenalis*, and *Iodamoeba buetschlii*. Due to the high morphological similarity between certain small protozoans in wet mount preparations, the software utilizes a “Miscellaneous Small Protozoans” class. This category aggregates organisms such as *Dientamoeba fragilis* trophozoites, *Chilomastix mesnili* trophozoites, *Endolimax nana* trophozoites, *Entamoeba hartmanni*, and *Iodamoeba buetschlii* trophozoites to maintain diagnostic sensitivity when specific taxonomic differentiation is visually overlapping. The operator review was blinded to previous traditional microscopy diagnosis of the same specimens, as the preparations were coded and anonymized. In addition, the operator has experience equivalent to that of microscopy experts who examine the original specimens for coproparasitological diagnosis.

### Data analysis

Statistical analyses were performed using Microsoft Excel 2016. Diagnostic positive percent agreement (PPA) and negative percent agreement (NPA) were calculated, and Cohen’s Kappa coefficient was employed to analyze the level of correlation between tests and qualitatively interpreted (14). PPA measures the proportion of positive results from the reference method that are also positive in the index test, while NPA measures the proportion of negative results that are also negative in the index test. PPA is considered equivalent to sensitivity, and NPA to specificity, and these measures are typically used when comparing a new diagnostic technique against a reference method that is not a perfect gold standard. Sample size was determined with 95% confidence considering known positive and negative specimens with Clopper–Pearson exact method. Discordant results between the AI methodology and traditional microscopy were described. Detection refers to the system’s ability to locate and distinguish a potential parasite from the background or artifacts, while identification refers to the accurate taxonomic classification of the detected object into a specific species or group. Diagnostic outcomes were categorized as follows: partial detection: identification of at least one etiology in a multi-pathogen specimen while missing others; artifacts: incorrectly identified objects as pathogens; false positives: microscopy-negative specimens categorized as positive by AI; and false negatives: microscopy-positive specimens categorized as negative by AI. Diagnostic gain was defined as any parasite or object detected by the AI that was previously missed by traditional microscopy.

We have classified the parasites as pathogenic and non-pathogenic following our national reference guidelines (11). *Giardia duodenalis*, *Entamoeba histolytica*, *Cystoisospora belli*, and helminth infections are considered pathogenic parasites, while *Blastocystis* spp. and *Dientamoeba fragilis* were considered controversial pathogenicity (11).

## RESULTS

### Diagnostic performance

Conventional microscopy examination was employed as the reference method to evaluate the diagnostic performance of the Human Fecal Ova & Parasite (O&P) Detection Wet Mount Iodine Solution (Techcyte). In addition, the operator review interpretation was included after AI-software detection (AI-system software + operator review). The AI-software correctly detected 109/147 (74.14%) positive specimens and 2/31 (6.45%) negative specimens. After operator review, performance diagnostic results improve in terms of PPA and NPA (Table 2). Moreover, two specimens were considered positive for (i) *Entamoeba* spp. and (ii) *Blastocystis* spp. and *Endolimax nana* by AI detection and operator review, which were previously considered negative by microscopic examination. The overall concordance between the AI system after operator review interpretation and the reference technique was 96.11%. The kappa coefficient indicates almost perfect agreement between microscopy and AI system after operator review. Regarding individual pathogen results (Table 3), the system demonstrated reliable identifications for most species. However, *Dientamoeba fragilis* specimens were on occasion misidentified as *Giardia duodenalis* and *Entamoeba* spp., and not inside the Miscellaneous Small Protozoans group. *Dicrocoelium dendriticum* and *Schistosoma intercalatum* were not categorized, therefore identified, by the software.

**TABLE 2:** Diagnostic performance of AI^*c*^-system software + operator review in comparison with microscopy examination gold standard method

	Gold standard (microscopy examination)				PPA^*a*^ (95% CI^*d*^)	NPA^*b*^ (95% CI^*d*^)	Kappa value (95% CI^*d*^)
AI^*c*^-system + operator review		Positive	Negative	Total	96.62 (91.88, 98.75)	93.33 (76.49, 98.84)	0.865 (0.767, 0.963)
	Positive	143	2	145			
	Negative	5	28	33			
	Total	148	30	178			

^*a*^ PPA, Positive Percent Agreement.

^*b*^ NPA, Negative Percent Agreement.

^*c*^ AI, artificial intelligence.

^*d*^ CI, confidence interval.

**TABLE 3:** Individual pathogen detection and identification concordance by AI-based system and AI-based system + operator review in comparison with traditional microscopy diagnosis^*f*^

Pathogen	Microscopic examination (gold standard)			Concordance of Techcyte detection	Concordance of Techcyte identification	Concordance after operator review	Additional comments
	Mono^*d*^	Mix^*e*^	Total				
*Blastocystis* spp.^*b*^	15	23	38	36/38 (94.73%)	36/36 (100%)	36/38 (94.73%)	
*Giardia duodenalis*	15	9	24	24/24 (100%)	24/24 (100%)	24/24 (100%)	
*Entamoeba coli*	11	8	19	19/19 (100%)	18/19 (94.73%)	19/19 (100%)	Identified as *Entamoeba* spp.^*b*^
*Entamoeba hartmanni*	1	1	2	2/2 (100%)	2/2 (100%)	2/2 (100%)	Identified as Misc.^*c*^ Small Protozoans and occasionally misclassified as *Endolimax nana*
*Entamoeba* spp.^*b*^	6	6	12	8/12 (66.66%)	8/8 (100%)	11/12 (91.67%)	
*Entamoeba histolytica/dispar*	6	5	11	9/11 (81.81%)	9/9 (100%)	11/11 (100%)	Identified as *Entamoeba* spp.^*b*^
*Endolimax nana*	10	2	12	11/12 (91.66%)	11/11 (100%)	11/12 (91.66%)	*Endolimax nana* trophozoites identified as Misc.^*c*^ Small Protozoans
*Dientamoeba fragilis*	19	8	27	23/27 (85.18%)	18/23 (78.23%)	23/27 (85.18%)	Identified as as Misc.^*c*^ Small Protozoans, and occasionally misclassified as *Giardia duodenalis* and *Entamoeba* spp.^*b*^
*Chilomastix mesnili*	1	2	3	3/3 (100%)	3/3 (100%)	3/3 (100%)	*Chilomastix mesnili* trophozoites identified as Misc.^*c*^ Small Protozoans
*Iodamoeba bütschlii*	5	0	5	2/5 (40%)	2/2 (100%)	4/5 (80%)	*Iodamoeba bütschlii* trophozoites identified as Misc.^*c*^ Small Protozoans
*Balantioides coli*	1	0	1	1/1 (100%)	1/1 (100%)	1/1 (100%)	
Negatives	NA^*a*^	NA^*a*^	31	2/31 (6.45%)	NA^*a*^	29/29 (100%)	Two specimens were considered positive after operator review
*Enterobius vermicularis*	4	1	5	5/5 (100%)	5/5 (100%)	5/5 (100%)	
*Schistosoma mansoni*	2	0	2	2/2 (100%)	2/2 (100%)	2/2 (100%)	
*Dicrocoelium dendriticum*	1	0	1	1/1 (100%)	NA^*a*^	1/1 (100%)	The *Techcyte* software does not have this classification category. Misclassified as *Paracapillaria philippinensis* and *Trichuris trichiura*
*Trichuris trichiura*	1	2	3	3/3 (100%)	3/3 (100%)	3/3 (100%)	
*Hymenolepis nana*	3	1	4	3/4 (75%)	3/3 (100%)	4/4 (100%)	
*Hookworm*	3	1	4	4/4 (100%)	4/4 (100%)	4/4 (100%)	
*Strongyloides stercoralis*	5	0	5	5/5 (100%)	5/5 (100%)	5/5 (100%)	
*Ascaris lumbricoides*	2	1	3	3/3 (100%)	3/3 (100%)	3/3 (100%)	
*Cystoisospora belli*	0	1	1	1/1 (100%)	1/1 (100%)	1/1 (100%)	
*Taenia* spp.^*b*^	2	0	2	2/2 (100%)	2/2 (100%)	2/2 (100%)	
*Diphyllobothrium latum*	1	0	1	1/1 (100%)	1/1 (100%)	1/1 (100%)	
*Fasciola hepatica*	1	0	1	1/1 (100%)	1/1 (100%)	1/1 (100%)	
*Hymenolepis diminuta*	1	0	1	1/1 (100%)	1/1 (100%)	1/1 (100%)	
*Schistosoma intercalatum*	0	1	1	0/1 (0%)	NA^*a*^	0/1 (0%)	The *Techcyte* software does not have this classification category

^*a*^ NA, not applicable.

^*b*^ spp.*,* species.

^*c*^ Misc., Miscellaneous.

^*d*^ Mono, monoinfection caused by a single microorganism.

^*e*^ Mix, mixed infection caused by two or more microorganisms.

^*f*^ *Detection concordance* refers to agreement between microscopic examination and Techcyte in the detection of an object, defined as the capture and display of an image by the system, regardless of subsequent identification. *Identification concordance* refers to agreement between both methods in the classification of the detected object as the same pathogen etiology. Differences in denominators reflect that identification concordance is calculated only for objects that were successfully detected and for which a definitive identification was possible. Concordance after operator review reflects agreement following human review of Techcyte results.

### Discordant specimens after AI-system software + operator review

A total number of 8/178 (4.49%) specimens were discordant or partially discordant after operator review involving the lack of detection of microorganism etiologies by the AI-system compared to conventional microscopy (Table 4).

**TABLE 4:** Discordant specimens in which the AI system failed to detect specific etiologies or microorganisms that had been previously identified by conventional microscopy.

Original diagnosis (conventional microscopy examination)	*Techcyte* + operator review diagnosis	Observations
*Blastocystis* spp.^*a*^ *Entamoeba* spp.^*a*^	*Blastocystis* spp.^*a*^	The specimen is considered POSITIVE although it is partially correct because *Entamoeba* spp.^*a*^ was not correctly identified
*Dientamoeba fragilis*	Negative	The specimen is considered NEGATIVE although it should be considered positive for *Dientamoeba fragilis* infection (false negative)
*Iodamoeba bütschlii*	Negative	The specimen is considered NEGATIVE although it should be considered positive for *Iodamoeba bütschlii* infection (false negative)
*Blastocystis* spp.^*a*^	Negative	The specimen is considered NEGATIVE although it should be considered positive for *Blastocystis* spp.^*a*^ infection (false negative)
*Dientamoeba fragilis* *Entamoeba coli* *Endolimax nana*	Negative	The specimen is considered NEGATIVE, although it should be considered positive for *Dientamoeba fragilis, Entamoeba coli, Endolimax nana* infection (false negative)
*Iodamoeba bütschlii*	*Blastocystis* spp.^*a*^	The specimen is considered POSITIVE for *Blastocystis* spp. although it should be considered positive for *Iodamoeba bütschlii* infection
*Dientamoeba fragilis*	Negative	The specimen is considered NEGATIVE although it should be considered positive for *Dientamoeba fragilis* infection (false negative).
*Schistosoma intercalatum* *Hookworm* *Trichuris trichiura*	*Hookworm* *Trichuris trichiura*	The specimen is considered POSITIVE although it is partially correct because *Schistosoma intercalatum* was not correctly identified

^*a*^ spp.*,* species.

The AI-based diagnostic system with operator review provided gains in the final diagnosis or objects that were not previously detected by the original microscopy examination results. After data analysis, there were a total of 74/178 (41.57%) specimens with additional parasite etiologies detected respect to conventional microscopy examination. In 2/178 (1.12%) specimens, the interpretation changed from negative using conventional microscopy to positive detection by the AI-based system. The most frequently detected microorganisms contributing to diagnostic improvements/gain were *Blastocystis* spp.*,* followed by *Endolimax nana*, *Entamoeba* spp., *Dientamoeba fragilis,* and *Entamoeba coli* (Table 5).

**TABLE 5:** Number of specimens with diagnostic gain after AI-system software + operator review, in comparison with traditional microscopy examination

Pathogen	Diagnostic gain
*Ascaris lumbricoides*	1
*Blastocystis* spp.^*a*^	48
*Chilomastix mesnili*	2
*Dientamoeba fragilis*	4
*Entamoeba coli*	4
*Entamoeba histolytica/dispar*	2
*Entamoeba* spp.^*a*^	8
*Endolimax nana*	16
*Giardia duodenalis*	2
*Iodamoeba buetschlii*	1
*Trichuris trichiura*	1

^*a*^ spp.: species.

Moreover, the AI-based system detected several objects within each specimen that were not accurately classified. These instances were subsequently flagged for expert review as part of the screening process. After the operator review, these objects were dismissed and considered as artifacts. Artifacts were detected in 162/178 (91.01%) specimens during raw analysis. The pathogens most frequently associated with false positive detections were *Chilomastix mesnili* 60/178 (33.7%), *Blastocystis* spp. 57/178 (32.0%), and *Giardia duodenalis* 52/178 (29.2%). Operator review was essential for accurate interpretation, to avoid diagnostic issues, and mainly to discard artifact detections. Before operator review, most of negative specimens 29/31 (93.54%) were considered positive by the AI-based software due to artifact misclassification.

### Reproducibility study of specimen volume

A total volume of 15 µL of concentrated stool specimen was placed on the microscope slide for image analysis. Specimen volume reproducibility study was performed using specimens (*n* = 8) with a very low helminth burden, and 10 replicate slides were prepared for each specimen to evaluate software detection. Results are shown in Table 6, with helminths detected in 49/78 (63.82%) low-parasitized specimen replicates.

**TABLE 6:** Number of detected objects by the *Techcyte* AI-system in low-parasitized helminth infected specimen replicates to evaluate the effectiveness of 15 µL volume for stool specimen analysis

Microscopy diagnosis	Rep^*a*^ 1	Rep^*a*^ 2	Rep^*a*^ 3	Rep^*a*^ 4	Rep^*a*^ 5	Rep^*a*^ 6	Rep^*a*^ 7	Rep^*a*^ 8	Rep^*a*^ 9	Rep^*a*^ 10
*Enterobius vermicularis*	1	2	3	0	3	2	3	1	1	2
*Hymenolepis nana*	0	2	0	0	0	0	0	0	0	2
*Enterobius vermicularis*	6	5	5	7	8	5	2	0	1	4
*Strongyloides stercoralis*	0	0	0	0	2	1	0	0	2	0
*Schistosoma mansoni*	1	0	0	0	0	0	0	0	0	0
*Hookworm*	6	5	6	1	3	5	1	4	2	1
*Schistosoma intercalatum*	2	3	1	1	1	1	4	1	1	0
*Trichuris* *trichiura*	7	3	1	2	1	6	0	2	0	2

^*a*^ Rep: specimen replicate on a different slide.

## DISCUSSION

Nowadays, there is an evident need to automate microscopy examination of stool specimens for intestinal parasites diagnosis in clinical laboratories. The increasing number of specimens and the associated workload pose a challenge for laboratory operators that requires substantial expertise and observational skill. Moreover, the time-consuming process of slide observation under a microscope can lead to fatigue-induced errors. In this study, the Human Fecal Ova & Parasite (O&P) Detection Wet Mount Iodine Solution (Techcyte) was clinically validated with promising results, showing a diagnostic gain in several specimens compared with conventional microscopic examination. The AI-based solution may serve as a suitable complement to support traditional microscopy and to increase sensitivity in low-parasitized specimens, enabling faster diagnosis (15). The results obtained in this study indicate that the proposed system should be regarded primarily as a screening tool to support conventional microscopy rather than as a definitive diagnostic method. The detections and identifications achieved by the CNN demonstrate its capacity to rapidly highlight structures of potential interest, thereby facilitating a pre-diagnostic assessment and potentially increasing the sensitivity of traditional microscopic examination. Nevertheless, expert review remains essential to confirm the findings and to exclude artifacts, and therefore, the system should be considered an assistive technology integrated within the routine laboratory workflow rather than a replacement for the final diagnostic interpretation. Moreover, Mathison et al. 2025 (16), developed and validated the mentioned AI-system with similar clinical validation results, and also observing additional detection of organisms in comparison with traditional microscopy (16).

Several studies have explored the development and application of deep neural networks for the detection of protozoa and helminths (17–19) although few have conducted clinical validation under real-world laboratory conditions. The FA280 fully automated digital feces analyser offers a simplified operation procedure, rapid detection speed, and an improved working environment for the detection of intestinal parasites (5). However, it incurs a higher cost per specimen and demonstrates lower sensitivity compared to the formalin-ethyl acetate concentration technique (5). As another example, SediMAX2 demonstrates 89.51% sensitivity and 98.15% specificity with 197 formol-fixed clinical stool specimens (20). Notably, in that study (20), the system functioned as an automated microscopic scanner to provide images for manual technician review, rather than performing fully autonomous identification. As an alternative to scanner-based methods, other studies have employed smartphone devices to perform soil-transmitted helminth AI-based diagnostics in resource-limited settings (20).

Our findings indicate that the automated microscopy system and AI-based *Techcyte* solution is configured to detect the maximum number of objects per specimen, which results in a high number of artifacts that can be easily discarded by the experienced microscopy operator. This configuration explains the low number of correctly identified negative specimens (2/31) initially, and the high proportion of correctly identified negative specimens after operator review (29/31). Low performance results for negative specimens due to artifact detection reaffirms the need to revise object detections by the operator. Fortunately, reclassification of these detections was relatively straightforward, due to the low number of artifacts detected per classification category (<5). It is noteworthy that the AI system demonstrated a capacity to detect organisms not included in its training set, such as *Dicrocoelium dendriticum*. This observation indicates that the model’s underlying convolutional architecture is capable of identifying morphological features that share latent characteristics with the trained classes, leading to cross-category activation. The “Misc. Small Protozoans” category was detected in 38/178 specimens although these were not considered as artifacts. The heterogeneity of objects in this category is diverse, as it is used to detect objects of interest without clear identification. Therefore, the system may be optimal in regions with low prevalence of intestinal parasites, where there is a high proportion of negative specimens that could be efficiently discarded by the AI-based method and the operator review.

Regarding other diagnostic techniques for coproparasitological analysis, microscopic examination remains as the reference technique. Methods such as antigen detection and molecular diagnostics are suitable although non-substitutive, alternatives to traditional microscopy (21, 22). Some rapid diagnostic tests have shown promising results for the identification of *Giardia intestinalis* and/or *Cryptosporidium* (23). However, the possibility of detecting different etiologies by antigen detection tests is limited in many cases. In the context of molecular testing for the clinical diagnosis of intestinal parasitic infections, several techniques have demonstrated high sensitivity and specificity compared to the reference microscopy (24). Moreover, some studies have shown that molecular techniques cannot completely replace microscopy, as they exhibit suboptimal yields for helminths diagnosis in fecal specimens and are costly as a result of the combination of syndromic panels (25). Correlating parasite burden with clinical significance remains challenging, as there is currently a lack of robust scientific evidence to support definitive thresholds for pathogenicity, although AI-microscopy could help to address this problem. It would be valuable to conduct a comparative analysis between artificial intelligence-based methods and molecular biology techniques, evaluating not only their diagnostic performance but also their overall cost-effectiveness.

Several limitations of the system were identified: (i) specimen stability and low volume for AI-based analysis and (ii) neural network specificity. Specimen stability is crucial to acquire representative digital images for AI analysis. While the morphology of pathogens is preserved in fixed stool specimens, the drying of the wet mount significantly degrades the overall scan quality. This loss of visual clarity and the resulting optical artifacts can hinder the accurate detection and identification of pathogens by the neural network, potentially leading to decreased PPA. With the scanner employed (analysis time per specimen: 2–3 minutes), a maximum of 15 specimens per load could be scanned without drying. If a higher number of specimens is added, the latter specimens end up drying out, thus affecting the images and therefore the diagnostic accuracy. Therefore, specimen drying and slide stability are limitations that can drastically impact reproducibility and PPA. While the specimen preparation is simple, affordable, and does not require highly qualified skills, a completely automated system from preparation to diagnosis would enhance efficiency (5). Other similar systems already offer such fully automated processes with Gram stain automated instruments (26). The concentrated specimen volume analyzed (15 µL) may also affect specimen drying in addition to the number of false negative specimens. As demonstrated in this study, helminthic infections with a low parasite burden may lead to a false negative result due to the volume of specimen used for the observation. Consequently, limit of detection studies would provide more information to set a proper sample volume for correct helminth detection. Additionally, while sample concentration is a standard requirement in parasitology, it becomes critical when using 15 µL volumes to counteract stochastic distribution and ensure biological representativeness, thereby preventing sensitivity loss in low-burden infections. In a context of low-prevalence settings, it is, therefore, important to be guided by other diagnostic parameters, such as eosinophilia or patient origin, and to consider using several replicates of a specimen to rule out this type of inaccuracy about helminth detection. Another limitation of our study is that most primary care specimens were examined using a single slide due to high workload, whereas a subset of specimens from Drassanes Vall Hebron included three replicates by conventional microscopy, which may have affected comparability between conventional and AI-assisted analysis. Finally, neural network specificity could be improved to reduce the operator review intervention. Although several forms were detected, some were misidentified or confused with stool artifacts. Neural network optimization by increasing image database and employing novel image analysis techniques, such as transformers or attention modules, could help to improve object detection metrics (27–29). Further research would be required to evaluate the introduction of this technology into routine care, assessing workflows and response times.

In conclusion, automated microscopy systems such as the Human Fecal Ova & Parasite (O&P) Detection Solution Wet Mount Iodine (Techcyte) offer promising support for traditional stool microscopy as a screening system. Despite its high PPA, the system’s detection of artifacts requires operator review for final diagnostic validation although object reclassification is straightforward. Enhancements in specimen preparation automation and combination with other diagnostic methods will ensure these systems become scalable tools for global use in diagnosing intestinal parasitic infections.

## Data Availability

Composite numerical data on which study calculations and results are based are included within the manuscript. Clinical metadata and individual patient results are not publicly available due to institutional policies and patient privacy considerations. The data sets used and/or analyzed during the current study are available upon reasonable request.
